# Regularization Solver Guided FISTA for Electrical Impedance Tomography

**DOI:** 10.3390/s23042233

**Published:** 2023-02-16

**Authors:** Qian Wang, Xiaoyan Chen, Di Wang, Zichen Wang, Xinyu Zhang, Na Xie, Lili Liu

**Affiliations:** 1 School of Electronic Information and Automation, Tianjin University of Science and Technology, Tianjin 300457, China; 2 College of Engineering, University of Alabama, Tuscaloosa, AL 35487, USA

**Keywords:** EIT, RS-FISTA, image reconstruction, inverse problem, FISTA

## Abstract

Electrical impedance tomography (EIT) is non-destructive monitoring technology that can visualize the conductivity distribution in the observed area. The inverse problem for imaging is characterized by a serious nonlinear and ill-posed nature, which leads to the low spatial resolution of the reconstructions. The iterative algorithm is an effective method to deal with the imaging inverse problem. However, the existing iterative imaging methods have some drawbacks, such as random and subjective initial parameter setting, very time consuming in vast iterations and shape blurring with less high-order information, etc. To solve these problems, this paper proposes a novel fast convergent iteration method for solving the inverse problem and designs an initial guess method based on an adaptive regularization parameter adjustment. This method is named the Regularization Solver Guided Fast Iterative Shrinkage Threshold Algorithm (RS-FISTA). The iterative solution process under the L1-norm regular constraint is derived in the LASSO problem. Meanwhile, the Nesterov accelerator is introduced to accelerate the gradient optimization race in the ISTA method. In order to make the initial guess contain more prior information and be independent of subjective factors such as human experience, a new adaptive regularization weight coefficient selection method is introduced into the initial conjecture of the FISTA iteration as it contains more accurate prior information of the conductivity distribution. The RS-FISTA method is compared with the methods of Landweber, CG, NOSER, Newton-Raphson, ISTA and FISTA, six different distributions with their optimal parameters. The SSIM, RMSE and PSNR of RS-FISTA methods are 0.7253, 3.44 and 37.55, respectively. In the performance test of convergence, the evaluation metrics of this method are relatively stable at 30 iterations. This shows that the proposed method not only has better visualization, but also has fast convergence. It is verified that the RS-FISTA algorithm is the better algorithm for EIT reconstruction from both simulation and physical experiments.

## 1. Introduction

Electrical Impedance Tomography (EIT) [1] is a noninvasive tomographic technique that visualizes the conductivity distribution in the measurement area [2]. A set of excitation electrodes attached to the boundary inject an alternating current to construct a sensitive field, and then the responding voltages are measured by other sensors sequentially. The conductivity distribution of the measurement region is reconstructed by an appropriate tomography algorithm. With the advantages of being non-damaging, noninvasive, non-radiation, low-cost, a simple operation and providing rich functional information, electrical impedance tomography has been researched deeply in the biomedical and industrial fields [3]. In biomedical applications, EIT has been used for lung imaging, which is based on the principle of assessing recoverable alveolar collapse and overdistension by images obtained during end-expiratory positive pressure titration [4,5]. In addition, EIT has also been used to varying degrees in brain function, brain activity and tumor localization studies [6,7,8]. EIT is more advantageous in long-time monitoring with functional visualizations compared to computed tomography (CT) and magnetic resonance imaging (MRI) [9,10]. In industrial applications, it is mainly used for process monitoring, allowing real-time monitoring of the gas or liquid transported in the pipeline and a more accurate estimation of the flow rate [11].

Currently, the core problem of solving the inverse problem of EIT, i.e., image reconstruction, is finding a suitable image reconstruction algorithm to obtain an accurate image to describe the conductivity parameters and boundary features. In order to solve this problem, many image reconstruction methods have been studied in two categories, iterative and non-iterative methods.

The non-iterative methods include the linear back projection method (LBP), truncated singular value decomposition (TSVD), the D-bar method, the Tikhonov regularization method, and the statistical shape constrained reconstruction method, etc. The back projection method (BP) is widely used as a basic solving method with the advantage of fast imaging. However, the linear back projection method (LBP) [12] and the improved filtered back projection method (FBP) [13] can reconstruct the approximate conductivity parameters containing serious artifacts and significant errors. The truncated singular value decomposition (TSVD) [14] method decomposes the system into singular values and uses partial singular value eigenvectors to solve the inverse problem, which reduces the influence of unknown parameters on the stability of the solution process. It improves the quality of FBP imaging to some extent, but the selection of the number of singular values and the eigenvectors often depends on the positive problem model and human experience, so it is not widely applicable to the scenario. The D-Bar method [15] is a direct method for solving nonlinear inverse problems, which converts the Laplace equation into the Schrödinger equation by nonlinear variable substitution. The D-Bar method utilizes the nonlinear Fourier transform to invert the distribution of the dielectric permittivity within the electric field using the exponential form of the solution. This method can improve the reconstruction quality under complex boundary conditions because it does not depend on the accuracy of the forward problem model and does not require an approximate solution of the Jacobian matrix. However, the calculation of the nonlinear transformation often requires the calculation of the DtN mapping, and it is built on the interrupted electrode model, so there are also certain artifacts on the ROI boundary. The regularization methods [16,17] are to impose a suitable penalty function to constrain the solution space under the convex optimization paradigm, such as the Tikhonov regularization method, which is based on L2-norm and is prone to cause boundary smoothing, which makes artifacts exist in the reconstructed images. Another regularization method is the total variation regularization method, which has the advantage of preserving boundary features but the proper regularization coefficient needs to be set by human experience [18]. The Statistical Shape Constrained Reconstruction method (SSCR) [19] is an ideal method that fully utilizes structural prior information as an optimization penalty function, where an accurate convergent solution is difficult to calculate considering the large condition number of the Jacobian matrix, alleviating the risk of inversing the ill-conditioned system matrix. However, the SSCR method requires strict Lipschitz continuity and is often limited by complex boundary conditions.

Iterative methods include the Landweber method, the Newton-Raphson method, the conjugate gradient method (CG), the soft threshold iterative method (Iterative Shrinkage-Thresholding Algorithm (ISTA), the NOSER (Newton’s one-step error reconstructor) method, the fast soft threshold method (Fast Iterative Shrinkage-Thresholding Algorithm, or FISTA), etc. Landweber’s algorithm [20] continuously reduces the error between the computed and real results by iterative methods. Since the Landweber algorithm needs to choose the appropriate regularization function to ensure the convergence of the solution, it often relies on the a priori information and empirically chosen regularization parameters, so there are sometimes artifacts interfering in the reconstruction results. The Newton-Raphson method [21,22] uses a quadratic function approximation instead of the objective function to find the extremum of this quadratic function and uses it as the approximation of the minima of the objective function. However, the Newton-Raphson method needs to calculate the Hessian matrix of the objective function for each iteration, which is computationally intensive when there are more variables. The conjugate gradient method (CG) [23] is a method for solving nonlinear systems of equations, which uses two eigenvectors in the conjugate direction to continuously iterate to search for the optimal solution of the EIT inverse problem and converges faster. However, the CG algorithm requires the system matrix to be strictly square, and the system matrix of EIT is an approximate result, so this approximation error is amplified in the solution, and the reconstruction results contain artifactual information, which cannot be applied to quantitative analysis scenarios. The NOSER (Newton’s one-step error reconstructor) method [24] requires only the Newton-Raphson method’s first step, setting an initial conductivity, to obtain an approximate solution of the field conductivity distribution. However, the image reconstructed in this way can only reflect the location and size of the medium, not the shape of the medium. The soft threshold iterative method (ISTA) [25,26] is an extension of the gradient descent method, and the iterative process only considers the information of the current point for gradient estimation to update the iteration point; the optimization process is in the shape of a zigzag towards the minimal value point, and the convergence speed is relatively slow. The fast soft threshold iteration method (FISTA) [27,28,29,30] adds a Nesterov acceleration technique to the ISTA method, which consists of a linear combination of the current point and the previous point and uses an acceleration technique to re-estimate the gradient and update the iteration points, which speeds up the algorithm. Due to the characteristics of ill-posedness and nonlinearity in the image reconstruction problem, it leads to the loss of some boundary information and makes the reconstructed images contain some artifacts. Although iterative algorithms have alleviated the effects of nonlinearity and ill-posedness on image reconstruction to a certain extent, the spatial resolution of images is still a subject that many researchers have devoted themselves to studying.

In summary, various iterative or non-iterative algorithms have different principles, but a single reconstruction algorithm is often used in specific applications. In this paper, we fully consider incorporating the non-iterative algorithm adaptive TR into the iterative FISTA algorithm to solve the problem of artificial empirical setting of the initial conductivity matrix in the FISTA algorithm, i.e., the Regularization Solver Guided Fast Iterative Shrinkage-Thresholding Algorithm (RS-FISTA for short). The algorithm first uses the adaptive regularization coefficient method to obtain the initial conductivity matrix of the FISTA. This paper adopts a fast soft thresholding image reconstruction strategy based on regularized solutions. The initial solution obtained by using the regularization method contains more a priori information, reduces the dependence on human settings, improves the imaging quality, and accelerates the convergence speed of the FISTA algorithm.

The contents of this paper are organized as follows. Section 2 is an overview of the EIT sensing model, the forward problem, and the inverse problem. Section 3 is the methodology of the proposed RS-FISTA algorithm. Section 4 briefly introduces the compared algorithm and the evaluation metrics. Section 5 presents the imaging results of the simulation and physical model, and compares the different algorithms quantitatively. Section 6 is the discussion and conclusions.

## 2. EIT Sensing Mechanism and Mathematical Model

### 2.1. EIT Sensing Model

**Figure 1:**
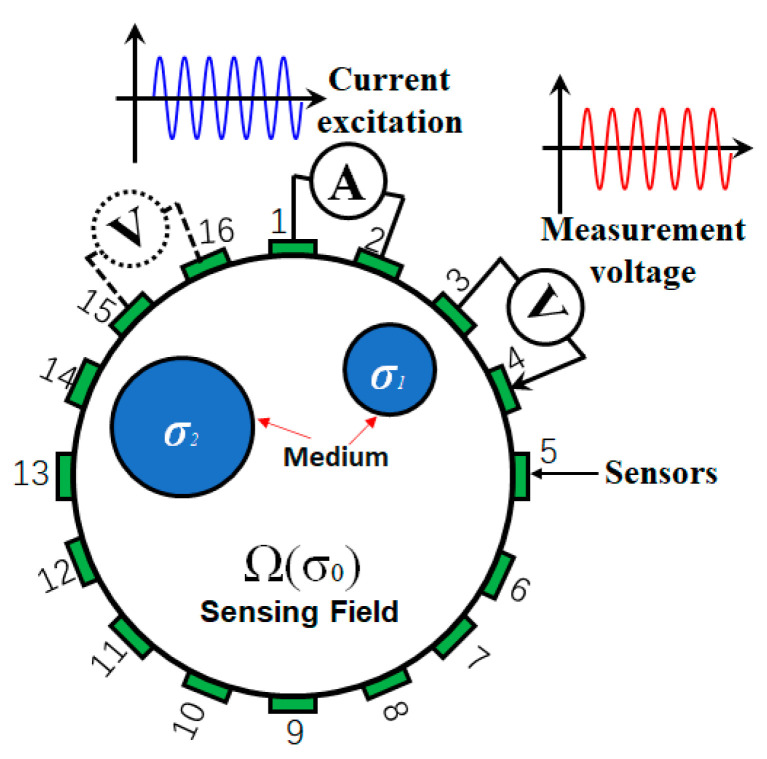
EIT simulation model measurement system.

Taking a 2D EIT sensing model as an example, the measurement system of EIT is shown in Figure 1. The data acquisition protocol of EIT adopts the adjacent current excitation adjacent voltage measurement [31]. A specific collection method is as follows: the current excitation is applied to one adjacent electrode pair as the initial excitation electrode (1–2 is selected as the first pair of the excitation current source in Figure 1), and then two adjacent electrodes are selected successively to measure voltages as the measurement electrode (3–4, 4–5, 5–6,……, 14–15, 15–16 in Figure 1). Using 16 pairs of adjacent electrodes as the excitation electrode successively, a total of 208 measurements are collected, which will be used as measurement describing one frame cross section.

In Figure 1, 1 to 16 represent 16 titanium electrodes attached equally on the boundary at the same height. Ω(σ_0_) represents the measurement area with conductivity σ_0_. σ_1_ and σ_2_ represent the conductivity of different inclusions. Ⓐ represents the current injected by a pair of adjacent electrodes. Ⓥ represents the measured voltage between a pair of adjacent electrodes.

In the EIT mathematical model, the electromagnetic field distribution in the region to be measured satisfies Maxwell’s equations [32], and its partial differential equation is as follows

(1) ∇×(σ(x)∇φ(x))=0,x∈Ω

where Ω is the measuring field, *σ*(*x*) and *φ*(*x*) represent the conductivity distribution and potential distribution in the measuring field, respectively. The unique solution of the differential Equation (1) can be obtained only under certain boundary conditions.

In the current excitation pattern, the response voltage at the boundary of the measured region is constrained by the Dirichlet boundary condition.

(2) $$\varphi (x)=u(x),x\in \partial \Omega \backslash \overset{16}{\underset{L=1}{\cup }}e_{L}$$

The boundary current density vector of the measurement area satisfies the Neumann boundary condition, and the equation is as follows

(3) $$\sigma (x)\frac{\partial \varphi (x)}{\partial n}=0,x\in \partial \Omega \backslash \overset{16}{\underset{L=1}{\cup }}e_{L}$$

where ***n*** is the outer normal vector on the boundary, and ∂Ω represents the boundary information of the measurement region.

Because the current density vector on the electrode surface is unknown, the differential form of Ohm’s law cannot be directly used on the electrode. Therefore, the boundary condition in the form of the integral is adopted, that is, on the electrode, the integral of the current density vector on the electrode surface is equal to the size of the injected current, which can be expressed as follows

(4) $$\int_{e_{L}}\sigma (x)\times \frac{\partial \varphi (x)}{\partial n}dS=I_{L},x\in e_{L},L=1,2,\cdots ,16$$

In EIT, the contact surface between the sensor and the measurement area usually has high impedance thin layers due to chemical reactions. The complete electrodes model (CEM) proposed by Somersalo et.al., is utilized [33,34,35]

(5) $$\varphi (x)+\rho_{L}\sigma (x)\times \frac{\partial \varphi (x)}{\partial n}=U_{L},x\in e_{L},L=1,2,\cdots ,16$$

where $\rho_{L}$ represents the contact impedance between the electrode and the contact surface, and *U_L_* represents the potential response at the *L* electrode.

Compared with the actual measurement, the boundary potential obtained by the CEM model with contact impedance has better consistency. To ensure the existence and uniqueness of the solution of Equation (1), it is necessary to guarantee the conservation of potential and charge in the EIT sensing model [36,37], namely

(6) $$\sum_{L=1}^{16}U_{L}=0$$

(7) $$\sum_{L=1}^{16}I_{L}=0$$

### 2.2. Forward Problem

Forward problem modeling methods include the finite element method (FEM) [38] and the boundary element method (BEM) [39]. The FEM divides the continuous measurement field into many discrete sub-areas (such as the triangle area), which can approximately represent the measuring field. The discrete form of the governing equation in Formula (1) is obtained by analyzing the conductivity and potential of each region, establishing the equations of each region, and combining them into a whole equation. Using FEM, the conductivity and voltage distribution in the measured field are written in discrete form as

(8) $$\sigma (x)=\sum_{i=1}^{N_{\sigma }}\sigma_{i}\phi (x),x\in \Omega$$

(9) $$\varphi (x)=\sum_{i=1}^{N_{\varphi }}\varphi_{k}\psi (x),x\in \Omega$$

where *σ*(*x*) represents the conductivity value in the subdivision grid, *φ*(*x*) represents the potential in the mesh, ϕ(*x*) represents the nodal basis function of the conductivity, *ψ*(*x*) represents the nodal basis function of the potential, and *Nσ* and *Nφ* represent the number of subdivision regions obtained by FEM. Using the standard Galerkin discretization [40], the solution of the direct problem can be approximated to the solution of a system of linear equations, respectively.

Below, *U*(*σ*) is used to indicate that σ discretely maps positively to *U*. The additive Gaussian model is used for measurement error, and the observation model is

(10) V=U(σ)+e

where V ∈ R^M*^^1^ represents the voltage value of the measured electrode, U(σ)∈R^M*1^ represents the direct problem operator (the solution of the direct problem is the calculated voltage), and e ∈ R^M*^^1^ represents the additive noise.

The inverse problem of EIT is the image reconstruction problem. The measurements and sensitivity matrix calculated from the forward problem are used to image the distribution of the parameters with conductivity. There exists a nonlinear relationship between the measurements and the conductivity distribution in the measurement field, as shown in Formula (10).

### 2.3. Inverse Problem

In general, the nonlinear functions that can be expanded using the Taylor series at some point can be approximated by linear combinations of the exponential function. Therefore, in the reference conductivity *σ*_0_, the Formula (11) can be obtained by the first order Taylor series expansion at

(11) $$U=U_{0}+A(\sigma )\times (\sigma -\sigma_{0})+O(\sigma )$$

where $A(\sigma )={\left.\frac{dU(\sigma )}{d\sigma }\right|}_{\sigma =\sigma_{0}}$ is the Jacobian matrix (matrix A ∈ R^M*N^), representing the differential of boundary voltage to conductivity. According to Geselowitz’s sensitivity theory [41], the Jacobian matrix is used to describe the changing relationship between measurements and the conductivity distribution, in which the elements are calculated as

(12) $$A_{ij}=-\int_{j^{\mathrm{th}}}^{}\nabla \varphi_{u}^{i}.\nabla \psi_{u}^{i}d\Omega$$

where *i* represents the electrode pair for the *i*-th adjacent current excitation adjacent voltage measurement, *j* represents the *j*-th pixel unit, $\nabla \varphi_{u}^{i}$ represents the gradient vector of the input current of the excitation electrode of the *i*-th pair in each electric field unit, and $\nabla \psi_{u}^{i}$ represents the gradient vector of the input current of the measurement electrode of the *j*-th pair in each electric field unit. After combining Formulas (11) and (12) and discrete approximation, the mathematical model of EIT inverse problem can be expressed as

(13) y=Ax+b

where *y = U − U*_0_, *x = σ − σ*_0_. *y* (matrix y ∈ R^M*^^1^) represents the difference between the boundary voltage value in different states and the boundary voltage value in the reference state. *x* (matrix x ∈ R^N*^^1^) represents the difference between the conductivity distribution in different states and the conductivity distribution in the reference state. *b* represents additive noise.

Since the mathematical model of the EIT inverse problem is ill-conditioned, Formula (10) cannot be uniquely solved and is usually calculated with the least squares error [42]. In order to prevent the least square problem from overfitting, a regularization term *R*(*x*) is added to the objective function, namely

(14) $$x=\underset{x}{\arg\min}\frac{1}{2}{\left\|Ax-y\right\|}_{2}^{2}+R(x)$$

## 3. Methodology

This section mainly introduces a new image reconstruction method, the RS-FISTA. The first part briefly introduces the FISTA method and analyzes the reconstruction process of the method. The second part briefly introduces the calculation method of regularization solver. The third part introduces the implementation process of the RS-FISTA.

### 3.1. FISTA Algorithm

When the regularization term R(x) uses the *L*_1_-norm constraint, the problem of Formula (14) can be converted into the LASSO problem (Least absolute shrinkage and selection operator, LASSO) [43], i.e.,

(15) $$\hat{x}=\underset{x}{\arg\min}\frac{1}{2}{\left\|Ax-y\right\|}_{2}^{2}+\lambda {\left\|x\right\|}_{1}$$

where *λ* is the regularization coefficient, and *λ* > 0. Let $f(x)=\frac{1}{2}{\left\|Ax-y\right\|}_{2}^{2}$, $g(x)={\left\|x\right\|}_{1}$.$g:{\mathbb{R}}^{N}\to \mathbb{R}$ is a continuous non-smooth convex function. $f:{\mathbb{R}}^{N}\to \mathbb{R}$ is a smooth convex function defined in ${\mathbb{C}}^{1,1}$, that is, a continuously differentiable function with Lipschitz continuous gradient, i.e., $\left\|\nabla f(x)-\nabla f(y)\right\|\leq L(f)\left\|x-y\right\|$, $x,y\in {\mathbb{R}}^{N}$. The smallest Lipschitz constants of ∇f is $L(f)=2\lambda_{\max}(A^{T}A)$.

#### 3.1.1. Non-Constrain LASSO Problem

Consider unconstrained optimization problems $\underset{x}{\min}\left\{F(x)\equiv f(x),x\in {\mathbb{R}}^{N}\right\}$. Normally, the gradient descent method

(16) $$x^{k+1}=x^{k}-t_{k}\nabla f(x^{k})$$

is used to solve the vector $x^{k+1}$. First, $f(x)=\frac{1}{2}{\left\|Ax-y\right\|}_{2}^{2}$ performs the second-order Taylor expansion,

(17) $$f(x)=\frac{1}{2}{\left\|Ax^{k}-y\right\|}_{2}^{2}+\langle A^{T}(Ax^{k}-y),x-x^{k}\rangle +\frac{1}{2t_{k}}{\left\|x-x^{k}\right\|}^{2}$$

Assuming f(x) satisfies the Lipschitz continuity condition, i.e., ∃L(f), s.t. $L(f)=\underset{x}{\sup}f^{\prime }(x)$. Then there exists any L≥L(f) with $f(x)=f(y)+\langle \nabla f(x^{k}),x-y\rangle +\frac{1}{2t_{k}}{\left\|x-y\right\|}^{2}$,$x,y\in {\mathbb{R}}^{N}$. At this time, in the neighborhood of any iteration point $x=x^{k}$, there is the following approximation

(18) $$f(x,x^{k})=f(x^{k})+\langle \nabla f(x^{k}),x-x^{k}\rangle +\frac{L}{2}{\left\|x-x^{k}\right\|}^{2}$$

In the next step, using Proxinal regularization method, i.e., the point where the function f(x) obtains the minimum value at $x=x^{k-1}$ is taken as the starting point of the next iteration $x=x^{k}$, then the optimization problem is

(19) $$x^{k}=\underset{x}{\arg\min}\left\{f(x^{k})+\langle \nabla f(x^{k}),x-x^{k}\rangle +\frac{1}{2t_{k}}{\left\|x-x^{k}\right\|}^{2}\right\}$$

where $t_{k}=\frac{1}{L}$. Equation (19) can be derived by the second-order Taylor expansion at $x=x^{k-1}$, where f(x) meets Lipschitz continuous constrain ${\left\|\nabla f(x^{k})-\nabla f(x^{k-1})\right\|}_{2}\leq L(f){\left\|x^{k}-x^{k-1}\right\|}_{2}$.

#### 3.1.2. Constrain LASSO Problem

When constraints are introduced, the optimization problem (15) can be written as $\underset{x}{\min}\left\{F(x)\equiv f(x)+g(x),x\in R^{N}\right\}$, then Equation (19) is rewritten as

(20) $$x^{k}=\underset{x}{\arg\min}\left\{f(x^{k-1})+\langle \nabla f(x^{k-1}),x-x^{k-1}\rangle +\frac{1}{2t_{k}}{\left\|x-x^{k-1}\right\|}^{2}+\lambda {\left\|x\right\|}_{1}\right\}$$

After ignoring the constant term in Equation (20), we can obtain

(21) $$x^{k}=\underset{x}{\arg\min}\left\{\frac{1}{2t_{k}}{\left\|x-(x^{k-1}-t_{k}\nabla f(x^{k-1}))\right\|}^{2}+\lambda {\left\|x\right\|}_{1}\right\}$$

Since the *L*_1_-norm is separable, the calculation of *x^k^* is simplified to solving the one-dimensional minimization problem for each of its components, which can be obtained through calculus

(22) $$x^{k}={Τ}_{\lambda t_{k}}[x^{k-1}-t_{k}\nabla f(x^{k-1})]$$

where ${Τ}_{\alpha }:{\mathbb{R}}^{N}\to {\mathbb{R}}^{N}$ is the shrinkage operator, which is defined as ${Τ}_{\alpha }(x_{i})=sign(xi)\times \max\{\left|xi\right|-a,0\}$, and α is the denoising parameter [44]. Literature [45] proves that the linear convergence rate of (21) is $O(\frac{1}{k})$. The expansion result of Equation (22) is

(23) $$x^{k}={Τ}_{\lambda t_{k}}[x^{k-1}-2t_{k}A^{T}(Ax^{k-1}-y)]$$

where *t_k_* is a suitable step size.

#### 3.1.3. Nesterov Accelerator

In order to speed up the convergence rate, the Nesterov accelerator is added to (23), where the calculation steps are as follows

(24) $$\left\{\begin{array}{l} x^{\left(k\right)}={Τ}_{\alpha }[z^{\left(k\right)}-\mu A^{T}(Az^{\left(k\right)}-y)]\\ t^{\left(k+1\right)}=\frac{1+\sqrt{1+4\times {\left(t^{\left(k\right)}\right)}^{2}}}{2}\\ z^{\left(k+1\right)}=x^{\left(k\right)}+\frac{t^{\left(k\right)}-1}{t^{\left(k+1\right)}}(x^{\left(k\right)}-x^{\left(k-1\right)}) \end{array}\right.$$

where *μ =* 1/*L* is a suitable step size, and *L* must be the upper bound of the maximum eigenvalue of *A^T^A* [46].

Literature [44] proves that, for any, there is

(25) $$F(x^{k})-F(x^{*})\leq \frac{2\beta L(f){\left\|x_{0}-x^{*}\right\|}^{2}}{{\left(k+1\right)}^{2}}$$

where β=1 is the constant search uber length, and β=η is the backtracking cruise step length. x* represents the solution when the algorithm convergence rate reaches $O(\frac{1}{k^{2}})$. Therefore, the convergence rate of this algorithm is increased from $O(\frac{1}{k})$ to $O(\frac{1}{k^{2}})$.

### 3.2. Regularization Solver (RS)

In the FISTA algorithm, the initial matrix *x*^(0)^ is selected as a fixed matrix and depends on human experience, which has great uncertainty. In this paper, based on the regularization idea, the solution can be solved by regularization, which can not only reduce the number of iterations, but also improve the solving accuracy of the iterative algorithm, so as to improve the spatial resolution of the image. Therefore, a penalty function based on the *L*_2_-norm constraint is used as the regularization factor in this paper, and its mathematical expression is

(26) $$R(\theta )={\left\|R(x-x_{0})\right\|}_{2}^{2}$$

where *R* is a particular continuous regularized matrix, and *x*_0_ is a hypothetical initial parameter based on the positive problem model. If *R = I* (identity matrix) and *x*_0_ = 0, the standard Tikhonov regularization mathematical expression of Formula (14) is

(27) $$\hat{x}=\underset{x}{\arg\min}\frac{1}{2}{\left\|Ax-y\right\|}_{2}^{2}+\lambda {\left\|Ix\right\|}_{2}^{2}$$

Define the objective function as

(28) $$f(x)=\frac{1}{2}{\left\|Ax-y\right\|}_{2}^{2}+\lambda {\left\|Ix\right\|}_{2}^{2}$$

Then the first-order differential of the objective function is

(29) $$\frac{df(x)}{dx}=\left(A^{T}A+\lambda I\right)x-A^{T}y$$

Let the first-order differential be zero, the optimal solution of the objective function can be expressed as

(30) $$\hat{x}={\left(A^{T}A+\lambda I\right)}^{-1}A^{T}y$$

where matrix A∈R^M*N^ represents the sensitivity matrix in the measurement field, and matrix $\hat{x}$ ∈ R^N*^^1^ represents the conductivity distribution in the measurement field, and matrix y ∈ R^M*^^1^ represents the potential distribution in the measurement field, and *λ^k^* represents the regularization coefficient.

In most cases, the regularization coefficient *λ* is evaluated according to the trial-and-error method or the “*L*” curve method, so the value taken out has great uncertainty. Therefore, a TR regularization method with adaptive regularization parameters is proposed in this paper. After each calculation of this method, the regularization coefficient *λ* will be adjusted according to the results of this (*x^k^*) and the expected (*y*), and then the new regularization parameters will be used to solve the solution of the next step, so that the value obtained is closer to the expected value. The formula for calculating the adaptive regularization coefficient *λ* can be expressed as

(31) $$\lambda^{k+1}=\lambda^{k}-\nabla_{x^{k}}{\left\|Ax^{k}-y\right\|}_{1}$$

So that $x^{k+1}={\left(A^{T}A+\lambda^{k+1}I\right)}^{-1}A^{T}y$. When the TR algorithm of adaptive regularization parameter iterates to an error less than 10^−2^, the initial matrix *x*^(0)^ required by the FISTA algorithm is obtained.

### 3.3. Pseudo-Code of RS-FISTA

The pseudo-code of this Algorithm 1 is as follows
**Algorithm 1** Regularization Solver Guided Fast Iterative Shrinkage Threshold Algorithm (RS-FISTA)INPUT: Sensitivity matrix *A*, voltage *y;* parameters: denoising parameter *α* = 10^−9^, relative tolerance *Epsilon* = 10^−5^, maximum iterations *Itermax*.
Setting the initial value *t*^(1)^ = 1, *k* = 1, *error* = 0Calculate the regular resolution as the initial value of the algorithm *x*^(0)^, *z*^(1)^ = *x*^(0)^Calculate the step size *μ* in the RS-FISTA method, $\mu =\frac{1}{L}$**If it** does not reach the convergence criterion or the error is greater than the relative tolerance,
(1)Calculate *x^(k)^*, $x^{\left(k\right)}={Τ}_{\alpha }[z^{\left(k\right)}-\mu A^{T}(Az^{\left(k\right)}-y)]$(2)Update parameter *t*^(^^*k* + 1)^ of the RS-FISTA method, $t^{\left(k+1\right)}=\frac{1+\sqrt{1+4\times {\left(t^{\left(k\right)}\right)}^{2}}}{2}$(3)Calculate $z^{\left(k+1\right)}$, $z^{\left(k+1\right)}=x^{\left(k\right)}+\frac{t^{\left(k\right)}-1}{t^{\left(k+1\right)}}(x^{\left(k\right)}-x^{\left(k-1\right)})$(4)Calculate *error*, $error=|y-Ax^{\left(k\right)}|$(5)k=k+1
    **end** OUTPUT: The calculated conductivity matrix *x*^(*k*)^, the error between the actual voltage and the estimated voltage error.

## 4. Comparison Algorithms and Evaluation Metrics

### 4.1. Comparison Algorithms

#### 4.1.1. Landweber Method

In EIT inverse problem, the conductivity distribution in the measurement field and the voltage at the electrode boundary are nonlinear problems. Moreover, the Landweber method is the most common method to solve nonlinear and ill-posed EIT problems [46]. The expression of the Landweber method is

(32) $$x_{k+1}=x_{k}+\lambda A^{T}(y-Ax_{k})$$

where $\lambda =2/\left(\lambda_{\max}+\lambda_{\min}\right)$ (*λ_max_* and *λ_min_* are the maximum and minimum positive special values of matrix *A^T^A*, respectively) [47]. In this article, the value of x0 is zero matrix.

#### 4.1.2. Conjugate Gradient Method (CG Method)

The CG method takes *x*_0_ as the initial conductivity matric, calculates the error *ω_0_ = y–Ax*_0_, gives the first search direction as *p*_0_ *= ω*_0_, and then carries out the iterative operation. The main iterative formula for this method is as follows

(33) $$\left\{\begin{array}{l} a_{k}=\frac{\left(\omega_{k},\omega_{k}\right)}{\left(Ap_{k},p_{k}\right)}\\ g_{k+1}=g_{k}+a_{k}p_{k}\\ \omega_{k+1}=\omega_{k}-a_{k}Jp_{k}\\ \beta_{k}=\frac{\left(\omega_{k+1},\omega_{k+1}\right)}{\left(\omega_{k},\omega_{k}\right)}\\ p_{k+1}=\omega_{k+1}-\beta_{k}p_{k} \end{array}\right.$$

where *k* = 1, 2, 3,…, n is the number of iterations, and the relationship between each corrected direction vector is conjugate, that is, *p_i_^T^Ap_j_ =* 0 (*I ≠ j*). In this article, the value of *x*_0_ is zero matrix.

#### 4.1.3. Iterative Shrink Threshold Algorithm (ISTA)

The ISTA method formula is shown in (15). The ISTA method solves Formula (15) through iterative Formula (34), which is shown below

(34) $$x^{\left(k+1\right)}={Τ}_{\alpha }[x^{\left(k\right)}-\mu A^{T}(Ax^{\left(k\right)}-y)]$$

where *μ* = 1/*L* is a suitable step size, and *L* must be the upper bound of the maximum eigenvalue of *A^T^A* [48], such as $L>\lambda_{\max}(A^{T}A)$. T_α_ is the shrink operator. For example, when $R(x)={\left\|x\right\|}_{1}$, the shrink operator is Tα(x)=soft(x,a), and $soft(x,a)=sign(xi)\times \max\{\left|xi\right|-a,0\}$. *α* is a denoising parameter. In this article, *µ* = 1/(*λ_min_* + *λ_max_*) and the value of *α* is 10^−9^. The initial matrix is zero matrix.

#### 4.1.4. NOSER Method

The NOSER method [24] is a fast static EIT algorithm based on the Newton method, but it does not require iteration and is only the first step of the Newton method, as shown in Formula (36)

(35) $$\left\{\begin{array}{l} E(x^{\left(0\right)})={\left\|y-Ax^{\left(0\right)}\right\|}^{2},\\ F(x^{\left(0\right)})=\frac{\partial E(x^{\left(0\right)})}{\partial x_{i}},i=1,2,\cdots ,N,\\ x=x^{\left(0\right)}-{\left[A(x^{\left(0\right)})\right]}^{-1}\times F(x^{\left(0\right)}). \end{array}\right.$$

where $x^{\left(0\right)}$ represents the initial conductivity matrix, $A(x^{\left(0\right)})$ represents the modified sensitivity matrix, and *N* represents the number of element divided in the measuring field. In this article, the value of $x^{\left(0\right)}$ is zero matrix.

#### 4.1.5. Newton-Raphson Method

The Newton-Raphson method [21] is an iterative method of unconstrained minimization for solving nonlinear functions. The basic idea of this method is to use the function approximation to replace the objective function, in order to find the minimum point of the quadratic function and take it as the approximate value of the minimum point of the objective function. The iterative formula of this method is shown in (36)

(36) $$x^{\left(k+1\right)}=x^{\left(k\right)}-{\left(A^{T}A+rI\right)}^{-1}A^{T}(Ax^{\left(k\right)}-y)$$

where *r* stands for regularization parameter and *I* stands for unit diagonal matrix. In this article, the value of *r* is 0.1.

### 4.2. Evaluation Metrics

The three main metrics of structure similarity (SSIM), root mean square error (RMSE) and peak signal to noise ratio (PSNR) are commonly used to quantify and evaluate image quality. SSIM is used to measure the similarity of two images. RMSE is used to measure the deviation between the calculated value and the expected value. PSNR is used to measure the degree of image satisfaction. The mathematical expression is as follows

(37) $$SSIM=\frac{2*\frac{1}{n}\sum_{i=1}^{n}rx_{i}\frac{1}{n}\sum_{i=1}^{n}rx_{i}{}^{*}\mathrm{cov}(rx,rx^{*})}{\left[{\left(\frac{1}{n}\sum_{i=1}^{n}rx_{i}\right)}^{2}+{\left(\frac{1}{n}\sum_{i=1}^{n}rx_{i}{}^{*}\right)}^{2}\right]{\mathrm{cov}}^{2}(rx,rx^{*})}$$

(38) $$RMSE=\sqrt{\frac{1}{nm}\sum_{j=1}^{m}{\sum_{i=1}^{n}\left[pix(j,i)-pix^{*}(j,i)\right]}^{2}}$$

(39) $$PSNR=10\log_{10}\frac{{\left(2^{k}-1\right)}^{2}}{RMSE^{2}}$$

where rx* represents the conductivity distribution of the reconstructed image, and *rx* is the conductivity distribution of the real image. pix* represents the pixel value of the reconstructed image, and *pix* represents the pixel value of the real image. 2*^k^* − 1 represents the maximum pixel value of the image.

In order to effectively evaluate the performance of the RS-FISTA method, this paper uses the rates at which average metrics are changing compared with those of other comparison methods (Landweber, CG, NOSER, Newton-Raphson, ISTA and FISTA methods), and the expression is

(40) $$CAM=\frac{\left|{AVE}_{RS-FISTA}-{AVE}_{x}\right|}{{AVE}_{x}}*100\%$$

where *AVE_RS-FISTA_* represents average metrics of the RS-FISTA method, and *AVEx* represents average metrics of the other comparison methods. The higher CAM means the better performances improved.

## 5. Experimentations and Results

The simulation experiments are implemented through the joint programming of MATLAB 2021a and COMSOL Multiphysics 5.6. The measurement field is triangulated by the finite element method (FEM) and obtains 3012 elements. Then the boundary voltage and sensitivity matrix of the field are obtained by using COMSOL Multiphysics 5.6 to solve the EIT forward problem. In the process of solving the EIT inverse problem, COMSOL Multiphysics and MATLAB are used to divide the measurement field into 64 × 64 mesh which could avoid inverse crime. During the simulation experiment, a tank with a radius of 0.095 m is set, and 16 electrodes, made of a Titanium material with 1 mm radius, are distributed on the wall of the tank at the same interval and height. A homogenous background with conducting is 1 S/m is set inside the tank, and the conductivity of the target object in the measurement field is 10^−12^ S/m. The excitation current amplitude is 4.5 mA, and the excitation current frequency is 100 kHz.

### 5.1. Simulation Experiments

**Figure 2:**
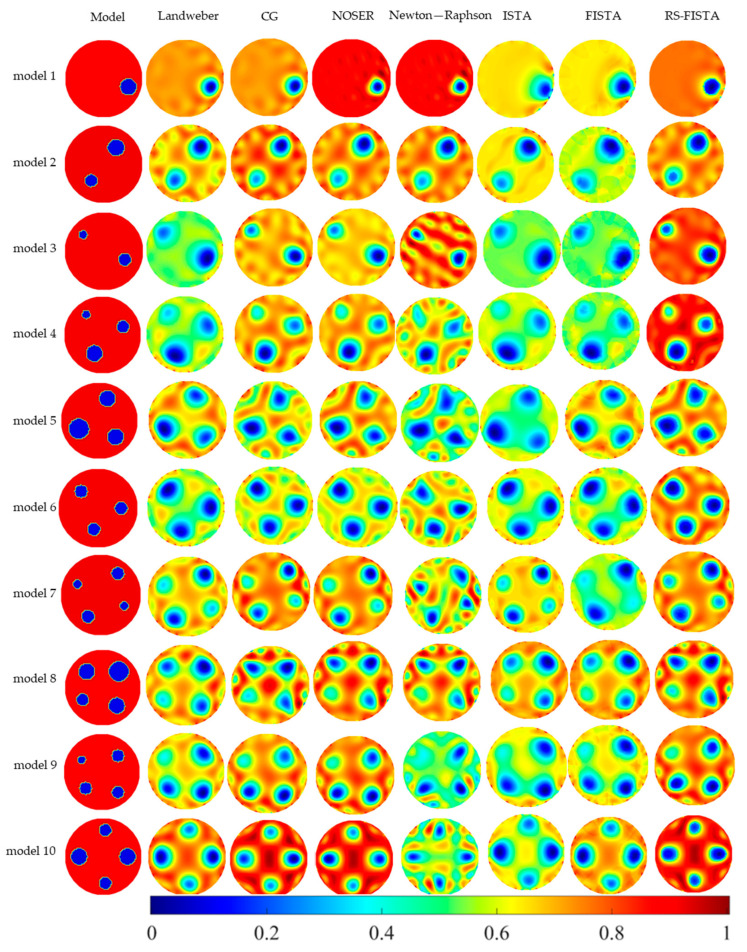
Simulation imaging results of different algorithms.

The results of reconstructed images obtained by the RS-FISTA method compare with those obtained by common Landweber, CG, NOSER, Newton-Raphson, ISTA and FISTA methods. The simulation imaging results are shown in Figure 2. From the results, we can see that the method has a better ability to deal with the nonlinearity of the EIT inverse problem.

It can be seen from Figure 2 that with the increase in the number of inclusions in the observation domain, the artifacts surrounding the objects in comparisons are not only more obvious with the smaller bubbles, but characteristics of the sharp boundary and the inclusion size are also significantly blurred. In contrast, due to the superiority of the RS-FISTA with the optimized regularization parameter, which could improve the quality in the initial guess with more prior information, the visualizations with more clear boundaries and more accurate conductivities are much better than the NOSER method and other optimized methods where the initial guess is manually set.

The results of image quantitative metrics, SSIM, RMSE, and PSNR, are shown in Table 1. It can be seen from the results of SSIM, RMSE and PSNR of the RS-FISTA method compared with the other six methods in Table 1 that the RS-FISTA method has significantly improved the image quality. The rates of average metrics (model 1~model 10) changing comparing with those of other comparison methods are shown in Table 1 (such as Formula (40)). It can be concluded that the SSIM of RS-FISTA method is 25.71% higher than the Landweber method, 11.41% higher than the CG method, 8.83% higher than the NOSER method, 27.84% higher than the Newton-Raphson method, 38.08% higher than the ISTA method and 33.37% higher than the FISTA method. The RMSE of the RS-FISTA method is 35.14% higher than that of the Landweber method, 10.21% higher than that of the CG method, 9.93% higher than that of the NOSER method, 29.76% higher than that of the Newton-Raphson method, 38.36% higher than that of the ISTA method, and 40.28% higher than that of the FISTA method. The PSNR of RS-FISTA method is 10.16% higher than that of the Landweber method, 2.49% higher than that of the CG method, 2.93% higher than that of the NOSER method, 8.35% higher than that of the Newton-Raphson method, 12.64% higher than that of the ISTA method, and 13.4% higher than that of the FISTA method.

### 5.2. Tank Experiments

**Figure 3:**
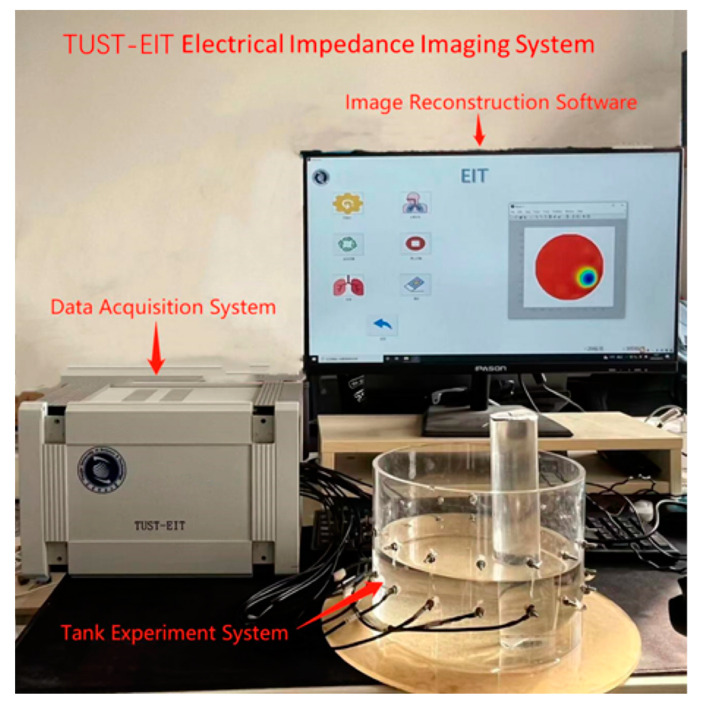
TUST-EIT Electrical Impedance Imaging System.

The boundary voltage signal acquisition equipment used in this paper is composed of the electrical impedance data acquisition system (EIT-DAS) and sensors attached on the tank. The system [49,50] is shown in Figure 3.

The DAS consists of a computer for data processing and image reconstruction, a data acquisition and signal processing module, and a tank with 16 titanium electrode sensors. In the experiment, 16 electrodes were distributed at the same interval and height on the tank (with a radius of 0.095 m) wall and uniform media (replaced by glass rods, and experimental data were obtained by changing the size, position, and number of objects) were placed in the tank. A proper concentration of NaCl was added to tap water to obtain the background conductivity of the experiment (the conductivity was set as 1 S/m), and the conductivity of the glass rod was approximately 0 S/m. During the experiment, the current with 4.5 mA amplitude and 100 kHz frequency is excited through the hardware. Then the voltage between the adjacent electrodes is measured to obtain the boundary voltage of the field. With the boundary voltage as the input value, image reconstruction was performed using the Landweber, CG, ISTA, FISTA, NOSER, Newton-Raphson and RS-FISTA methods. The result of image reconstruction is shown in Figure 3.

**Figure 4:**
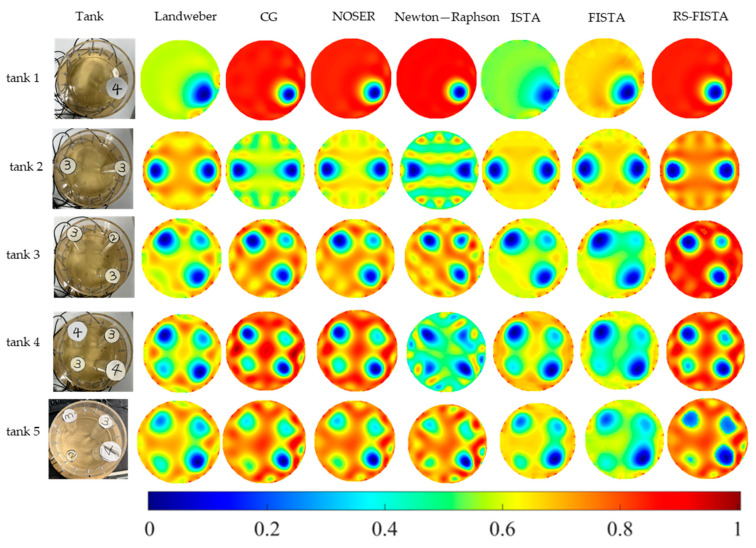
Imaging results of bucket experiment with different algorithms (the numbers 2–4 represent the diameter of the inclusions).

It can be seen from Figure 4, using other methods (such as Landweber, CG, NOSER, Newton-Raphson, ISTA and FISTA), with the increase of the number of inclusions in the observation domain, the position information of inclusions with small geometric diameter in the reconstructed image is more blurred, and the “transition area” between the background and the include is larger. However, because the RS-FISTA method uses the initial guess with prior information, the artifacts are less in reconstructions. So, compared with other optimized methods where the initial guess is manually set, this method can better display the location information of inclusions with a small diameter.

The results of image quantitative metrics, SSIM, RMSE, PSNR, are shown in Table 2. It can be seen from the results of SSIM, RMSE and PSNR of the RS-FISTA method compared with the other six methods in Table 2 that the RS-FISTA method has significantly improved the image quality. The rates of average metrics (tank 1~tank 5) changing compared with those of other methods are shown in Table 2 (such as Formula (40)). It can be seen from the comparison chart of evaluation indicators that the SSIM of RS-FISTA method is 24.26% higher than that of the Landweber method, 11.07% higher than that of the CG method, 9.38% higher than that of the NOSER method, 24.01% higher than that of the Newton-Raphson method, 35.80% higher than that of the ISTA method, and 38.16% higher than that of the FISTA method. The RMSE of the RS-FISTA method is 36.73% higher than that of the Landweber method, 21.08% higher than that of the CG method, 10.65% higher than that of the NOSER method, 36.83% higher than that of the Newton-Raphson method, 46.05% higher than that of the ISTA method, and 44.57% higher than that of the FISTA method. The PSNR of RS-FISTA method is 12.12% higher than that of the Landweber method, 4.66% higher than that of the CG method, 2.38% higher than that of the NOSER method, 9.29% higher than that of Newton-Raphson method, 16.31% higher than that of the ISTA method, and 14.87% higher than that of the FISTA method.

### 5.3. Noise Experiments

**Figure 5:**
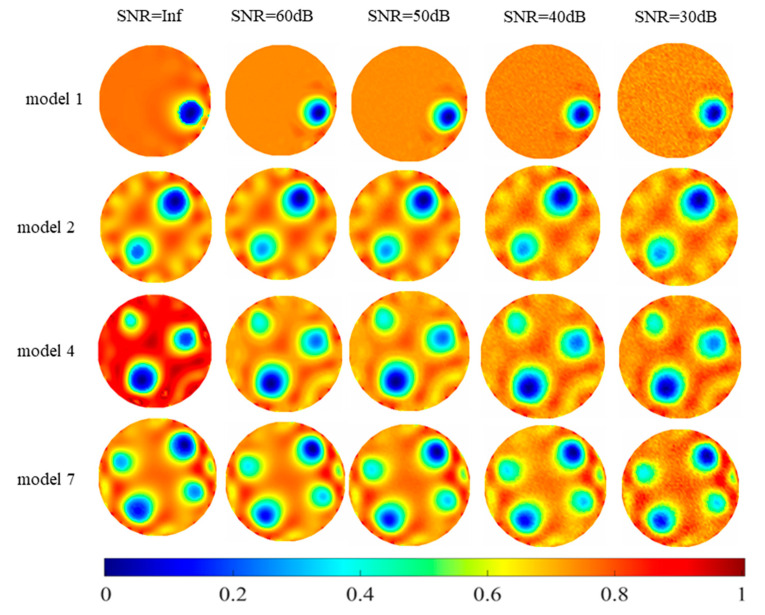
Image reconstruction results of noise experiment.

In order to test the robustness of the RS-FISTA method to noise with different signal to noise ratios (SNRs), different levels of Gaussian white noise (SNR = 60 dB, 50 dB, 40 dB, 30 dB) were added to the simulation experiment. In the experiment, models with different numbers were selected as experimental samples. The image reconstruction results of the experiment are shown in Figure 5.

It can be seen from the figure that when the RS-FISTA method’s SNR = Inf, 60 dB and 50 dB, the reconstructed image can better express the distribution of the inclusions in the original measurement field, and the reconstructed image has less artifact information. As the level of white Gaussian noise decreases, the position information of the inclusions begins to become blurred, but the position information of the inclusions can still be better expressed. In summary, it shows that the robustness of the RS-FISTA method is good, and even if the input signal contains noisy interference, the image reconstructed by the algorithm still has a good visualization effect.

## 6. Discussion and Conclusions

### 6.1. Experiments Analysis

In this paper, the results of RS are taken as the initial values, so that the initial values of the iterative method are closer to the optimal solution of the objective function. Therefore, the RS-FISTA method is used for image reconstruction. From the visual image, we can see that the reconstructed image has less artifacts, relatively accurate location information and clearer boundaries. From the quantitative metrics, we can see that the value of SSIM is improved, indicating that the shape information saved is relatively good. The value of RMSE is reduced which indicates that the distribution of conductivity is relatively accurate. The value of PSNR is improved, which shows that the visualization effect of the image is relatively good. In a word, the reconstructed image has good spatial resolution.

### 6.2. Discussion on Convergence Iterations

**Figure 6:**
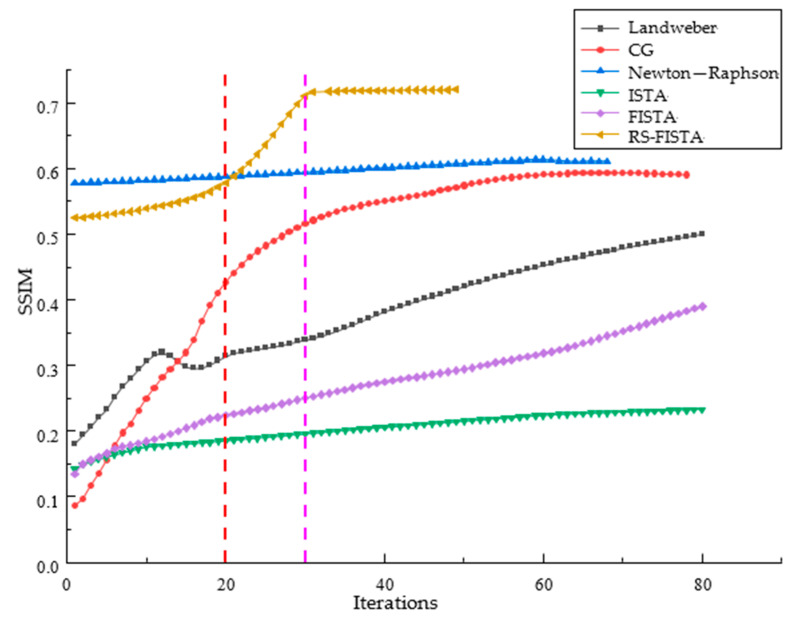
The SSIM variation with iterations of different methods (Use red and purple vertical lines to divide the number of iterations into three zones).

In order to verify the convergence speed of the RS-FISTA method in this paper, except that NOSER is a one-step iterative imaging method, the other six methods (Landweber, CG, ISTA, FISTA, Newton-Raphson and RS-FISTA) successively perform different times of iterative operations on the physical experimental model and compare the SSIM of the reconstructed images. The SSIM of the reconstructed images of the six imaging methods are shown in Figure 6.

It can be observed that within 20 iterations, the SSIM index of the RS-FISTA is only less than that of Newton-Raphson, but better than other methods. From 20 to 30 iterations, the SSIM index of the RS-FISTA increases rapidly, which is distinctly higher than the other five methods. After 30 iterations, the SSIM index of the RS-FISTA has a stable performance in SSIM and superior to the other methods. If you discount the complexity of different methods, the RS-FISTA method gives a satisfactory convergent efficiency.

The initial guess of the FISTA method is usually set according to experience, which has great uncertainty. The RS-FISTA method takes the result of the adaptive regularization solution as the initial guess. This has the advantage that the initial guess is closer to the optimal solution of the objective function, thereby reducing the number of iterations of the method. This method also introduces Nesterov acceleration technology. This acceleration technique first realizes an acceleration in the *x^k^* direction by the linear combination of one of the previous two points {*x^k^, x^k^*^−1^}, and then accelerates in the gradient direction of the function through the contraction operator, so that the method can quickly solve the optimal solution of the objective function. Other algorithms only accelerate in the gradient direction, so this method has faster iteration speed and fewer iterations than other methods.

### 6.3. Conclusions

In this paper, the RS-FISTA method is proposed to solve the problems of excessive artifact information and poor visualization effect in electrical impedance tomography. In this method, the TR regularization of the adaptive parameters is calculated as the initial value of the input, and the Nesterov acceleration technique is used to solve the problem of slow convergence of the reconstructed image method effectively. In this paper, the corresponding experiment is carried out, and the imaging effect and image evaluation metrics of the tank experiment are analyzed. The results show that compared with the methods of Landweber, CG, ISTA, FISTA, Newton-Raphson and NOSER, the reconstructed images not only have less artifact information and a better visualization effect, but also effectively improve the convergence speed of the method.

## Figures and Tables

**Table 1:** Imaging evaluation metrics of simulation experiments of different methods.

		Model 1	Model 2	Model 3	Model 4	Model 5	Model 6	Model 7	Model 8	Model 9	Model 10	Average	CAM (%)
SSIM	Landweber	0.6314	0.6560	0.4610	0.4755	0.6318	0.4950	0.5780	0.6106	0.5571	0.6958	0.5792	**25.71**
	CG	0.7396	0.7060	0.6627	0.6503	0.5920	0.5912	0.6285	0.6054	0.6220	0.7377	0.6535	**11.41**
	NOSER	0.8159	0.6872	0.6269	0.6481	0.6546	0.5969	0.6261	0.6311	0.6505	0.7533	0.6691	**8.83**
	Newton-Raphson	0.8139	0.6885	0.6484	0.5559	0.4951	0.6009	0.5134	0.5920	0.4122	0.3751	0.5695	**27.84**
	ISTA	0.5407	0.6064	0.4544	0.4734	0.4639	0.5068	0.5947	0.6011	0.4810	0.5506	0.5273	**38.08**
	FISTA	0.6776	0.5293	0.4572	0.4707	0.6241	0.4898	0.4177	0.5998	0.5315	0.6617	0.5459	**33.37**
	RS-FISTA	0.8642	0.8186	0.6824	0.7682	0.7002	0.6709	0.6467	0.6618	0.6629	0.8052	**0.7281**	
RMSE	Landweber	4.1512	3.9204	9.5336	8.0922	4.7712	8.0247	5.0658	4.4982	5.9477	4.6288	5.8634	**35.14**
	CG	2.2004	3.1769	3.3918	4.1313	5.5283	6.4158	4.3224	4.8471	4.7803	4.5498	4.3344	**10.21**
	NOSER	3.0971	3.5265	3.9107	4.2672	4.4006	6.4183	4.2285	4.1526	4.5111	4.6980	4.3211	**9.93**
	Newton-Raphson	3.0886	3.5107	3.2570	5.7159	7.5509	5.8777	5.9001	4.4714	8.8449	7.1195	5.5337	**29.67**
	ISTA	3.5797	4.4090	9.6882	7.1962	7.7374	7.0151	4.6817	4.6917	7.3017	6.8406	6.3141	**38.36**
	FISTA	3.5157	6.3535	9.6369	8.8076	4.8842	7.6574	8.5180	4.4623	6.4900	4.8457	6.5171	**40.28**
	RS-FISTA	2.0716	3.1538	3.2188	4.1123	4.3155	4.8655	4.1900	4.0350	4.4323	4.5241	**3.8919**	
PSNR	Landweber	35.76700003	36.2641	28.5456	29.9695	34.5583	30.0423	34.0379	35.0700	32.6438	35.4034	33.2302	**10.16**
	CG	41.2807	38.0908	37.5221	35.8091	33.2789	31.9858	35.2599	34.4212	34.5417	34.971	35.7161	**2.49**
	NOSER	38.3118	37.1838	36.2858	35.528	35.2607	31.9824	35.6071	35.7645	35.0451	34.6925	35.5662	**2.92**
	Newton-Raphson	38.3356	37.2229	37.8745	32.9891	30.5708	32.7466	32.7137	35.1219	29.1969	31.0818	33.7854	**8.35**
	ISTA	37.0539	35.2440	28.4059	30.9887	30.3589	31.2102	34.7228	34.7042	30.8623	31.429	32.4980	**12.64**
	FISTA	37.2105	32.0705	28.4521	29.2336	34.3549	30.4492	29.5240	35.1396	31.8860	34.4236	32.2744	**13.42**
	RS-FISTA	41.8048	38.1542	37.9769	35.8490	35.4301	34.3883	35.6865	36.0140	35.1982	35.5628	**36.6065**	

**Table 2:** Experimental evaluation metrics for imaging results of 7 different methods.

		Tank 1	Tank 2	Tank 3	Tank 4	Tank 5	Average	CAM (%)
SSIM	Landweber	0.5958	0.6597	0.5288	0.5767	0.5574	0.5837	**24.26**
	CG	0.7985	0.587	0.6081	0.6905	0.581	0.653	**11.07**
	NOSER	0.802	0.6365	0.6042	0.68	0.5929	0.6631	**9.38**
	Newton-Raphson	0.8064	0.5174	0.6532	0.3932	0.5542	0.5849	**24.01**
	ISTA	0.4397	0.6265	0.5055	0.5657	0.533	0.5341	**35.8**
	FISTA	0.6857	0.6027	0.442	0.4566	0.4379	0.525	**38.16**
	RS-FISTA	0.8188	0.7125	0.679	0.7166	0.6996	**0.7253**	
RMSE	Landweber	6.2126	4.2844	5.9118	5.5837	5.202	5.4389	**36.73**
	CG	2.3693	7.1053	4.579	3.8017	3.9482	4.3607	**21.08**
	NOSER	2.3117	4.588	4.7049	3.7014	3.9506	3.8513	**10.65**
	Newton-Raphson	2.4178	8.1639	4.0846	8.5454	4.0266	5.4477	**36.83**
	ISTA	9.506	4.7078	6.1617	5.7965	5.7223	6.3789	**46.05**
	FISTA	3.3496	5.0391	7.1245	7.6213	7.9063	6.2082	**44.57**
	RS-FISTA	2.3031	3.6883	4.0452	3.5559	3.6139	**3.4413**	
PSNR	Landweber	32.2653	35.4931	32.6965	33.1924	33.8074	33.4909	**12.12**
	CG	40.6386	31.0991	34.9153	36.5313	36.2029	35.8774	**4.66**
	NOSER	40.8521	34.8983	34.6797	36.7636	36.1975	36.6784	**2.38**
	Newton-Raphson	40.4623	29.8928	35.9077	29.4961	36.032	34.3582	**9.29**
	ISTA	28.5709	34.6745	32.3367	32.8675	32.9795	32.2858	**16.31**
	FISTA	37.631	34.0838	31.0757	30.4902	30.1713	32.6904	**14.87**
	RS-FISTA	40.8844	36.7942	35.992	37.1118	36.9713	**37.5507**	

## Data Availability

No new data are created.
